# An Attenuated Recombinant Newcastle Disease Virus of Genotype VII Generated by Reverse Genetics

**DOI:** 10.3390/v17121618

**Published:** 2025-12-15

**Authors:** Hongze Pang, Yidan Bo, Jiawei Chen, Yongzhi Xue, Baishi Lei, Kuan Zhao, Yu Huang, Wenming Jiang, Wuchao Zhang, Wanzhe Yuan

**Affiliations:** 1 College of Veterinary Medicine, Hebei Agricultural University, Baoding 071001, China; 2 Hebei Veterinary Biotechnology Innovation Center, Hebei Agricultural University, Baoding 071001, China; 3 Fujian Key Laboratory for Prevention and Control of Avian Diseases, Institute of Animal Husbandry and Veterinary Medicine, Fujian Academy of Agricultural Sciences, Fuzhou 350000, China; 4 National Reference Laboratory for Newcastle Disease, China Animal Health and Epidemiology Center, Qingdao 266000, China; 5 North China Research Center of Animal Epidemic Pathogen Biology, China Agriculture Ministry, Baoding 071001, China

**Keywords:** newcastle disease virus, genotype VII, reverse genetics, attenuated, vaccine candidate

## Abstract

Genotype VII Newcastle disease virus (NDV) has been confirmed as the predominant epidemic strain in China. Traditional vaccine strains fail to provide complete immune protection when challenged with an epidemic strain. NDV vaccines with phylogenetic relationships closer to those of the endemic viruses demonstrate improved protective efficacy in reducing viral shedding and transmission. This research seeks to develop attenuated vaccine strains that are specifically aligned with NDV genotype VII. A reverse genetics system for the genotype VII NDV HB strain was developed, successfully rescuing the attenuated recombinant virus aHB by substituting the fusion protein (F) cleavage site motif “^112^R-R-Q-K-R↓F^117^” with “^112^G-R-Q-G-R↓L^117^.” Recombinant aHB virus attenuation was verified by assessing the mean death time (MDT) and intracerebral pathogenicity index (ICPI). The attenuated aHB strain demonstrated greater proliferation titers than did the virulent HB and rHB strains both in vivo and in vitro. Furthermore, the genome exhibited significant genetic stability even after 10 passages in chicken embryos. When challenged with the HB strain of NDV genotype VII, the aHB-inactivated vaccine provided 100% protection to chickens and effectively prevented viral shedding. These findings indicate that recombinant aHB may serve as an effective vaccine candidate.

## 1. Introduction

Newcastle disease (ND), an avian illness caused by the ND virus (NDV), is extremely contagious and pathogenic, spreading across the globe and causing considerable economic harm to the poultry industry [1,2]. When chicken flocks are infected with Newcastle disease, they typically exhibit symptoms, including high fever, respiratory distress, diarrhea, mucosal hemorrhage, neurological symptoms, and decreased egg production [3,4]. NDVs are members of the genus *Orthoavulavirus*, the subfamily *Avulavirinae*, species *Orthoavulavirus javaense* in the family *Paramyxoviridae* [5]. This virus is an RNA virus with an envelope that features a non-segmented, single-stranded, negative-sense RNA genome. Its genome length can be 15,186, 15,192, or 15,198 nucleotides, following the “rule of six [6,7,8].” The NDV genome consists of six genes arranged in the order 3′-*NP-P-M-F-HN-L*-5′, which code for the nucleocapsid protein (NP), phosphoprotein (P), matrix protein (M), fusion protein (F), hemagglutinin–neuraminidase (HN), and large protein (L) [9,10]. Through RNA editing, the NDV *P* gene can produce two non-structural proteins, V and W [11,12,13]. The leader sequence is located at the 3′ end of the viral genome, and the trailer sequence is at the 5′ end, both containing regulatory signals crucial for transcription and replication [14]. The leader and trailer sequences were highly conserved, measuring 55 and 114 nt, respectively.

Multiple pathogenicity tests are employed to evaluate NDV virulence, including the mean death time (MDT) in embryonated eggs, the intravenous pathogenicity index (IVPI) in six-week-old chickens, and the intracerebral pathogenicity index (ICPI) in one-day-old chickens [15]. ICPI is the internationally recognized system for assessing NDV virulence. NDV strains are categorized into three types of pathogenicity: velogenic (1.5 < ICPI < 2; MDT < 60 h), mesogenic (0.7 < ICPI < 1.5; 60 h <MDT < 90 h), and lentogenic (ICPI < 0.7; MDT > 90 h), based on differences in virulence [16,17]. To date, all NDV isolates have been identified as belonging to a single serotype. The antigens of different NDV strains vary; thus, NDVs are classified into multiple genotypes [18]. Based on a comparative analysis of the *F* gene sequences, NDV strains were classified into two categories: Class I and Class II. Class I contained only one genotype (genotype 1), which was further subdivided into three subgenotypes: 1.1.1, 1.1.2, and 1.2. These are primarily low-virulence NDV strains isolated from wild birds [19]. Conversely, Class II NDVs exhibit greater genetic diversity, currently comprising 21 genotypes (I–XXI) that are further subdivided into multiple subtypes. These include both low and highly pathogenic NDV strains isolated from poultry and wild birds [20,21]. Most outbreaks of ND worldwide have been associated with pathogenic NDV strains belonging to Class II genotypes V, VI, VII, and IX [22]. In China, NDV genotype VII is the dominant strain, whereas highly pathogenic NDV genotypes VI, VIII, IX, and XII have been reported sporadically [23,24,25,26].

Reverse genetics has facilitated recombinant NDV creation for studying NDV virulence, developing vaccines, and exploring their oncolytic uses. In 1999, Peeters et al. successfully employed reverse genetics to rescue the initial rNDV [27]. Numerous NDV strains have been successfully rescued using the positive-sense anti-genome RNA [28]. Additionally, Sendai virus (SeV) has utilized cDNA clones with negative-sense genome RNA for viral rescue [29]; nonetheless, no such reports currently exist for NDV [30]. Moreover, using cDNA clones of positive-stranded inverted-genome RNA resulted in higher rescue efficiency during viral rescue experiments [30]. The T7 polymerase and cell RNA polymerase II systems are widely used for virus rescue. However, our laboratory was unsuccessful in attempting to rescue NDV using the cell RNA polymerase II system. In this study, we employed the T7 polymerase system. Several cell lines have been reported to stably express T7 RNA polymerase, with the BSR T7/5 cell line being the most widely used. This cell line required adding G418 during passaging for cell selection and maintenance. Notably, Römer-Oberdörfer et al. first used this cell line for NDV rescue [31]. Subsequently, several teams have rescued NDV using BSR T7/5 cells [32,33,34,35,36].

The increase in the host range and susceptibility of animal species to genotype VII NDV makes it essential to develop vaccines that can effectively tackle genotype VII NDV [37,38,39,40]. This research seeks to develop attenuated vaccine strains that are specifically aligned with NDV genotype VII. The NDV HB strain is a highly pathogenic subtype VII.1.1. Based on this HB strain, we established a reverse genetics system using the T7 polymerase system and performed genetic modifications on the HB strain. The recombinant attenuated aHB virus was rescued using helper plasmids encoding *NP*, *P*, and *L* genes from the LaSota strain, and its potential as a vaccine candidate was examined.

## 2. Materials and Methods

### 2.1. Virus, Cells, and Vectors

NDV strain HB, a velogenic genotype VII, was isolated in 2016 from diseased chickens with ND at a poultry farm in Hebei Province (the full-length genome sequence of the HB strain is detailed in the Supplementary Materials). The HB strain used in this study is distinct from the NDV strains [MK611802] and [MK342603] in the DNA database and exhibited an MDT of 45 h and an ICPI of 1.92. The ground tissue suspension was inoculated into 10-day-old specific pathogen-free (SPF) chicken embryos (Beijing Boehringer Ingelheim Vital Biotechnology Co., Ltd., Beijing, China) for virus isolation. The virus was purified from chicken embryo fibroblast cells and cultured in 10-day-old embryonated SPF chicken eggs. BSR T7/5 cells stably expressing the phage T7 RNA polymerase were provided by Dr. Wenming Jiang of the China Animal Health and Epidemiology Center. The cells were maintained in Dulbecco’s modified Eagle’s medium (DMEM; Gibco, Carlsbad, CA, USA) supplemented with 10% fetal calf serum and 1 mg/mL G418 Sulfate (Gibco, Carlsbad, CA, USA). DF-1 cells were maintained in DMEM supplemented with 10% fetal calf serum. The vectors pOK12, pEGFP-N1, and pCI-neo were gifts from Dr. Wenming Jiang. pCI-LaNP, pCI-LaP, pCI-LaL, and minigenome plasmid pOK-Lamini of the NDV strain LaSota were stored at the Veterinary Biological Products Laboratory of Hebei Agricultural University (these plasmids were constructed by Yidan Bo).

### 2.2. Construction of Expression Plasmids and Phylogenetic Analysis

**Figure 1:**
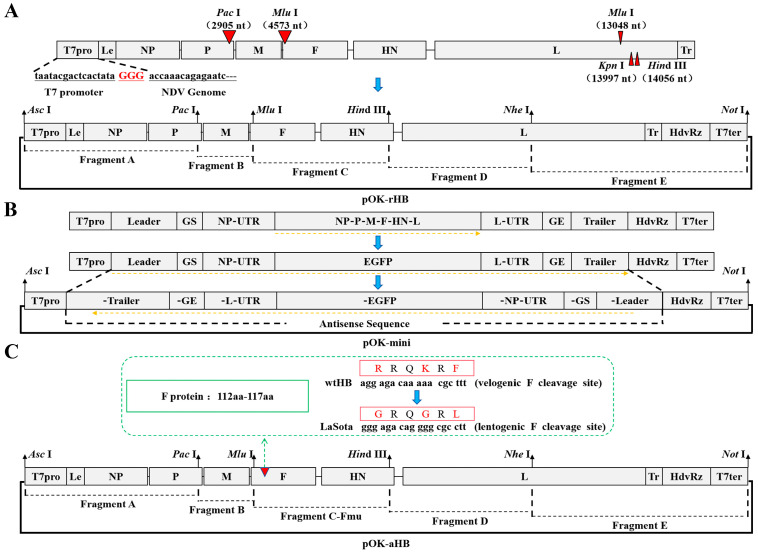
Schematic diagram of the construction of plasmids pOK-rHB, pOK-mini, and pOK-aHB. (**A**) Development of a complete genomic plasmid for the NDV strain HB. Le represents the leader sequence, and Tr indicates the trailer sequence. The red triangle indicates the location where the gene-directed mutation is performed. T7pro represents the sequence of the T7 promoter, and T7ter identifies the sequence of the T7 terminator. The “GGG” addition could enhance the transcription from the T7 promoter. The extra “GGG” sequence transcribed from cDNA was not added to the rescued NDV RNA genome. HdvRz denotes the hepatitis delta virus ribozyme site, ensuring the transcription product adheres to the “rule of six.” (**B**) Construction of the minigenome plasmid for the NDV HB strain. GS represents the gene-start signal, and GE is the gene-end signal. (**C**) Complete genomic plasmid development for the attenuated HB strain. The red triangle indicates that the mutation occurred at amino acids 112–117 of the F protein.

The complete plasmid of the HB strain was constructed by splitting the entire cDNA sequence into five parts (A–E). The 5′ end of segment A was altered to incorporate an AscI restriction site, a T7 promoter sequence, and an added “GGG” nucleotide sequence. The “GGG” addition could enhance the transcription from the T7 promoter. The 3′ end of the genome was modified by inserting the hepatitis delta virus ribozyme (HdvRz) sequence, followed by the T7 terminator sequence, with a NotI restriction site added at the terminator’s 3′ end. Genomic cDNA was directionally modified using synonymous mutations as genetic markers that did not alter the original amino acid sequence. The restriction enzyme sites KpnI, MluI, and HindIII were removed from the *L* gene, whereas PacI and MluI were added to the *P* and *F* genes. The original T7 promoter sequence in the pOK12 vector was removed, and the vector was renamed pOK12-M. Subsequently, gene fragments A–E were cloned into pOK12-M. The complete plasmid of the HB strain was designated pOK-rHB (Figure 1A). Primers for identifying each of the above mutation sites are provided in the Supplementary Materials.

The *NP*, *P*, and *L* open reading frames were amplified from the pOK-rHB plasmid. Kozak sequences were added to the 5′ end of each gene before cloning them into the pCI-neo vector, resulting in the creation of helper plasmids pCI-NP, pCI-P, and pCI-L.

To evaluate helper plasmid usefulness, a minigenome plasmid was constructed. The plasmid’s coding region for the “*NP-P-M-F-HN-L*” gene was substituted with the *enhanced green fluorescent protein (EGFP)* gene. The minigenome was cloned in antisense orientation into pOK12-M, resulting in the creation of pOK-mini (Figure 1B).

Sequence alignment of the *F* and *HN* genes for HB strain, LaSota strain, B1 strain, and other genotype VII NDV strains was performed using the ClustalW program in Lasergene software version 5.0 (DNASTAR, Madison, WI, USA). A phylogenetic tree was constructed based on the complete *F* gene coding sequence (1–1662 nt) using the neighbor-joining method in MEGA 11 (Mega Limited, Auckland, New Zealand), with 1000 bootstrap replicates.

### 2.3. Effects of Different Helper Plasmids on EGFP Expression in the Minigenome

Helper plasmids from different strains may have different functional activities. To investigate the functional activities of helper plasmids in different strains, minigenome plasmids and helper plasmids from the HB and LaSota strains were cross-used.

BSR T7/5 cells underwent three culture cycles in DMEM supplemented with G418. The day before transfection, 4 × 10^5^ BSR T7/5 cells were plated in 6-well plates and cultured until they reached 80–90% confluence. Cells were transfected with plasmids using Lipofectamine 2000 (Invitrogen, Carlsbad, CA, USA) after replacing DMEM with serum-free Opti-MEM (Gibco, Carlsbad, CA, USA). Lipofectamine 2000 (2 μL) was utilized with 1 μg of DNA. Four hours later, the transfection medium was replaced with 2 mL of DMEM supplemented with 10% fetal calf serum. EGFP-expressing cell counts were assessed by fluorescence microscopy at 24 h post-transfection.

### 2.4. rHB Strain Virus Rescue

BSR T7/5 cells underwent three culture cycles in DMEM supplemented with G418. The day before transfection, 4 × 10^5^ BSR T7/5 cells were plated in 6-well plates and cultured until they reached 80–90% confluence. BSR T7/5 cells were transfected with a 10 μg DNA mixture comprising pOK-rHB, pCI-LaNP, pCI-LaP, and pCI-LaL in a 4:2:1:1 ratio. Six hours later, the transfection medium was replaced with 2 mL of DMEM supplemented with 10% fetal calf serum. Three days later, the cell supernatants were collected, and the monolayer cells were harvested after incubation with trypsin. The cells were centrifuged at 1000× *g* for 5 min and resuspended in 1 mL of the collected medium. To amplify the recovered viruses, 200 μL of the cell samples were inoculated into 10-day-old SPF chicken embryos. The allantoic fluid was collected from chicken embryos after 4 days, and the hemagglutination (HA) titer was measured using a 96-well microtiter plate. Genetic markers of the *L* gene were sequenced and analyzed using the Lasergene software version 5.0 (DNASTAR, Madison, WI, USA).

### 2.5. HB Virus Attenuation

The cleavage site of the F protein in the HB virus was modified from ^112^R-R-Q-K-R↓F^117^ to ^112^G-R-Q-G-R↓L^117^ to achieve attenuation. Gene fragment C was inserted into plasmid pOK12, and the F protein cleavage site was directionally mutated using overlapping polymerase chain reaction (PCR) technology. The C-Fmu fragment was substituted with an equivalent segment from the full-length pOK-rHB cDNA (Figure 1C). The cDNA clone containing the mutated *F* gene was designated as pOK-aHB. BSR T7/5 cells (4 × 10^5^) were seeded in 6-well plates and cultured to 80–90% confluence before transfection. BSR T7/5 cells were transfected with a 10 μg DNA mixture comprising pOK-rHB, pCI-LaNP, pCI-LaP, and pCI-LaL in a 4:2:1:1 ratio. Six hours post-transfection, the medium was replaced with 2 mL DMEM supplemented with 10% fetal calf serum and 1 µg/mL TPCK-treated trypsin (Sigma-Aldrich, St. Louis, MO, USA).

The collected cell mixtures were inoculated into 10-day-old SPF chicken embryos for viral recovery. The allantoic fluid was collected from chicken embryos after 4 days, and the HA titer was measured using a 96-well microtiter plate. The F protein cleavage site was sequenced and analyzed using Lasergene software version 5.0 (DNASTAR).

### 2.6. Stability and Biological Properties of the Generated Viruses

Genetic stability of the modified cleavage site in the *F* gene and other markers in the *L* gene was assessed by serially passaging the recombinant viruses rHB and aHB 10 times in 10-day-old embryonated eggs. Reverse-transcription polymerase chain reaction (RT-PCR) sequenced and analyzed the cleavage sites in the *F* gene and other genetic markers in the *L* gene of the virus from passages 5 and 10 (the primer set used to identify the F protein cleavage site sequence is presented in the Supplementary Materials).

Standard procedures for MDT, 50% egg infectious dose (EID_50_), and ICPI were conducted to assess the pathogenicity of recombinant viruses in vivo. The in vitro pathogenicity of the recombinant viruses was evaluated by assessing the cytopathic effect induced by the rescued NDVs in BSR T7/5 cells.

### 2.7. Evaluation of Immunoprotective Effect of the aHB-Inactivated Vaccine

Recombinant aHB was inactivated using β-propiolactone (Solarbio, Beijing, China) at 0.5 mL/L under 37 °C for 2 h. After inactivation, the virus was stored at 4 °C. Subsequently, the inactivated virus was combined with an aqueous adjuvant (Montanide^TM^ Gel P, Seppic Shanghai Chemical Specialities Co., Ltd., Shanghai, China) at a 9:1 ratio. The inactivated vaccine was prepared after thorough agitation to ensure homogeneity.

Thirty 21-day-old SPF chickens (Beijing Boehringer Ingelheim Vital Biotechnology Co., Ltd., Beijing, China) were randomly divided into three groups, with ten chickens in each group. Group 1 was administered 0.5 mL of the inactivated aHB vaccine, while Groups 2 and 3 received 0.5 mL phosphate-buffered saline (PBS). Serum samples were collected at 7, 14, and 21 days post-vaccination to measure hemagglutination inhibition (HI) antibody titers. Twenty-one days after immunization, Groups 1 and 2 were administered a 0.2 mL intranasal/intraocular inoculation of the NDV HB strain (10^5^ EID_50_), while Group 3 received 0.1 mL PBS as the negative control. Clinical symptoms were observed daily for 14 consecutive days after the challenge. On days 3, 5, 7, and 10 post-challenge, throat and cloacal swabs were collected to determine viral shedding in the flocks. The swab samples were processed in PBS containing penicillin-streptomycin, and the supernatant was tested for HA activity in 10-day-old SPF chicken embryos.

### 2.8. Statistical Analysis

Statistical analyses and graphing were performed using GraphPad Prism software (version 8.0; GraphPad Software, Inc., San Diego, CA, USA). *p* values of <0.05 were considered statistically significant; *p* values of <0.001 were considered extremely significant.

## 3. Results

### 3.1. Genetic Evolution Analysis of the F Gene in HB Strain

**Figure 2:**
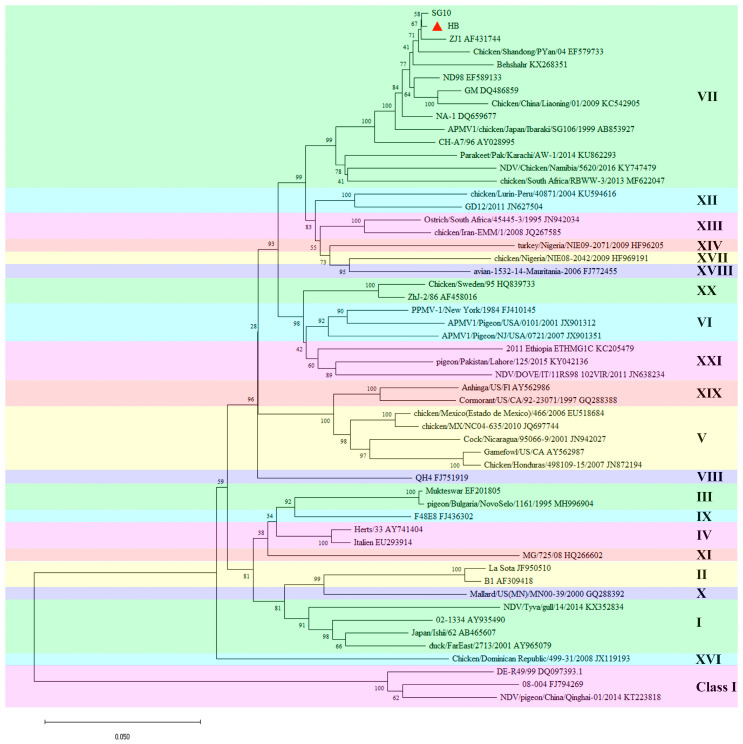
Evolutionary analysis of the complete *F* gene sequence of HB strain. The tree was constructed using the neighbor-joining method in the MEGA 11 software. The analysis involved 54 nucleotide sequences. The tree was drawn to scale, with branch lengths measured in the number of substitutions per site. The value on the node represents the bootstrap value. The red triangle indicates the strain HB used in this study.

Phylogenetic analysis revealed that the complete *F* gene sequence of HB strain was classified as genotype VII of NDV strains in Class II (Figure 2). The F protein’s basic cleavage site amino acid sequence was ^112^R-R-Q-K-R↓F^117^.

Sequence alignment analysis demonstrated that the *F* gene of the HB strain shared 84.8% and 85.1% homology with the LaSota and B1 strains, respectively, while showing 88.3–99.6% homology with other genotype VII NDV strains. The *HN* gene of the HB strain demonstrated 82.1% and 82.2% homology with the LaSota and B1 strains, respectively, and 89.2–99.5% homology with other genotype VII NDV strains. Therefore, there were significant differences between the HB and classical vaccine strains in the major virulence genes *F* and *HN*.

### 3.2. Functional Activity of Different Helper Plasmids

**Figure 3:**
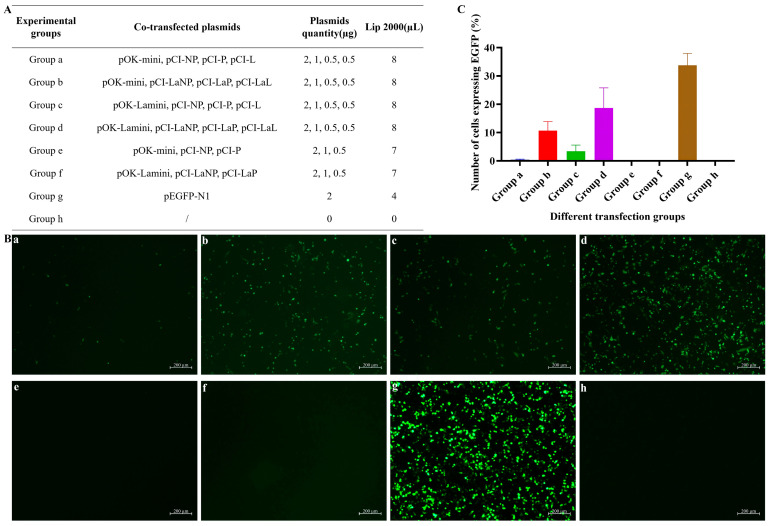
Minigenome assay with helper plasmids and minigenome plasmids from different strains. BSR T7/5 cells were transfected with various plasmid combinations, and EGFP expression was examined using a fluorescence microscope after 24 h. (**A**) Combination strategy for helper plasmids and minigenome plasmids. (**B**) (**a**) Plasmids pOK-mini, pCI-NP, pCI-P, and pCI-L were used to co-transfect BSR T7/5 cells. (**b**) Plasmids pOK-mini, pCI-LaNP, pCI-LaP, and pCI-LaL were used to co-transfect BSR T7/5 cells. (**c**) Plasmids pOK-Lamini, pCI-NP, pCI-P, and pCI-L were used to co-transfect BSR T7/5 cells. (**d**) Plasmids pOK-Lamini, pCI-LaNP, pCI-LaP, and pCI-LaL were used to co-transfect BSR T7/5 cells. (**e**) Plasmids pOK-mini, pCI-NP, and pCI-P were used to co-transfect BSR T7/5 cells. (**f**) Plasmids pOK-Lamini, pCI-LaNP, and pCI-LaP were used to co-transfect BSR T7/5 cells. (**g**) Transfection with the plasmid pEGFP-N1 was used as the positive control. (**h**) No plasmid was transformed as a negative control. (**C**) A relative quantitative analysis was performed in order to determine the number of cells expressing EGFP across all transfection groups under the same observation conditions.

Plasmids were co-transfected in different combinations (Figure 3A). The antisense *EGFP* gene in the minigenome plasmid requires the cooperative action of the NP, P, and L proteins for its expression. Twenty-four hours post-co-transfection of the plasmids into BSR T7/5 cells, distinct green fluorescence was observed under fluorescence microscopy across groups a–d (Figure 3B(a–d)). Contrastingly, Groups e and f, which lacked helper plasmids pCI-L and pCI-LaL, respectively, exhibited no green fluorescence under fluorescence microscopy (Figure 3B(e,f)). BSR T7/5 cells transfected solely with pEGFP-N1 exhibited substantial green fluorescence, indicating successful transfection (Figure 3B(g)), thereby suggesting that EGFP expression depends on the interaction between the NP, P, and L proteins. When rescue was performed using the LaSota strain helper plasmids pCI-LaNP, pCI-LaP, and pCI-LaL, Groups b and d exhibited significantly more green fluorescence than did Groups a and c. This indicates that under the transfection conditions established in this study, the helper plasmids from the LaSota strain exhibited superior functional activity compared to those from the HB strain, enabling a greater number of BSR T7/5 cells to successfully express EGFP.

### 3.3. Recombinant Virus rHB Construction

**Figure 4:**
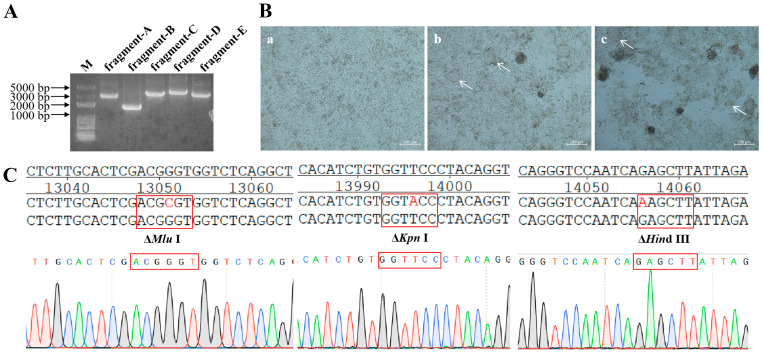
Construction and characterization of the recombinant virus rHB. (**A**) RT-PCR products of gene fragments A-E from the NDV HB strain. (**B**) (**a**) No plasmid was transformed as a negative control. (**b**) After co-transfection of plasmids pOK-rHB, pCI-LaNP, pCI-LaP, and pCI-LaL for 96 h, cytopathic effects were observed in BSR T7/5 cells. (**c**) After co-transfection of plasmids pOK-rHB, pCI-LaNP, pCI-LaP, and pCI-LaL for 120 h, cytopathic effects were observed in BSR T7/5 cells. The location indicated by the white arrow demonstrates the cytopathic effect. (**C**) Genetic markers of the recombinant virus strain rHB. The restriction enzyme sites MluI, KpnI, and HindIII were eliminated.

Gene fragments A–E were amplified and sequentially cloned into the pOK12-M vector. The results of 1% agarose gel electrophoresis confirmed the successful amplification of all fragments. As expected, their sizes were 2913, 1681, 3479, 3711, and 3465 bp, respectively (Figure 4A).

BSR T7/5 cells were transfected with 10 μg of plasmids pOK-rHB, pCI-LaNP, pCI-LaP, and pCI-LaL. A small syncytial cytopathic effect was observed 96 h post-transfection. As the culture time increased, a significant syncytial cytopathic effect was observed at 120 h post-transfection (Figure 4B).

The transfected cell mixture was introduced into 10-day-old SPF chicken embryos. Embryos were incubated for 4 d before harvesting the allantoic fluid. The HA titer of the virus in the allantoic fluid was determined to be 2^5^ (32) HA. Sequencing of genetic markers in the *L* gene revealed that three restriction enzyme sites were successfully knocked out through the targeted mutations, and the rHB strain was successfully rescued (Figure 4C).

### 3.4. Recombinant Virus aHB Construction

**Figure 5:**
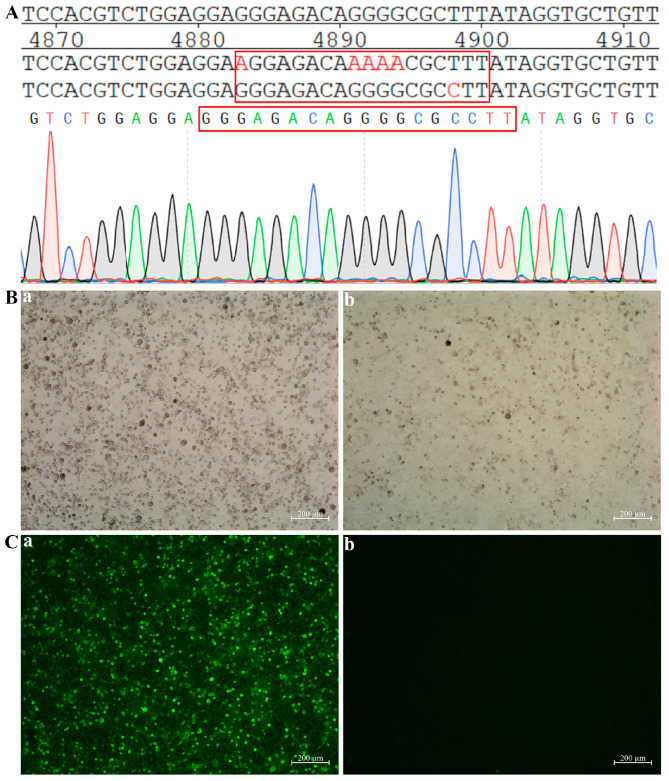
Construction and characterization of the recombinant virus aHB. (**A**) Identification of the cleavage site sequence in the F protein of the recombinant aHB strain. (**B**) (**a**) Cytopathic effects induced by attenuated strain aHB after infection of BSR T7/5 cells. The cells were photographed at 96 h post-infection (hpi). (**b**) BSR T7/5 cells not infected with the virus were utilized as negative controls. (**C**) (**a**) Replication of recombinant virus aHB in BSR T7/5 cells was evaluated using an indirect immunofluorescence assay. (**b**) BSR T7/5 cells not infected with the virus were utilized as negative controls. BSR T7/5 cells were cultured in medium supplemented with TPCK-treated trypsin (1 µg/mL).

The F protein cleavage site in the pOK-rHB plasmid was altered from ^112^R-R-Q-K-R↓F^117^ to ^112^G-R-Q-G-R↓L^117^ to create the full-length pOK-aHB plasmid for attenuated virus rescue. BSR T7/5 cells were transfected with 10 μg of plasmids, including pOK-aHB, PCI-LaNP, PCI-LaP, and PCI-LaL. Interestingly, no cytopathic effects were observed 120 h post-transfection. By day 5 post-inoculation of the transfected supernatant into embryonated SPF eggs, HA-positive allantoic fluid with a 4 log_2_ HA titer was obtained, confirming the successful rescue of the mutant aHB virus. Sequencing results of the F protein cleavage site revealed that the aHB strain was successfully rescued (Figure 5A).

BSR T7/5 cells were cultured in medium supplemented with TPCK-treated trypsin (1 µg/mL). Viral aHB was introduced into BSR T7/5 cells, and no cytopathic effects were detected after 96 h (Figure 5B). The aHB infection status was detected via indirect immunofluorescence using chicken-derived polyclonal antibodies against NDV. Abundant green fluorescence was observed under fluorescence microscopy, indicating that although aHB inoculation did not induce cytopathic effects, it could still rapidly infect the cells (Figure 5C).

### 3.5. Biological Characteristics of the Recombinant Viruses

As per World Organization for Animal Health (OIE) standards, the biological characteristics of recombinant viruses were assessed by measuring MDT, EID_50_, and ICPI. Virulent strains are defined by the OIE as viruses that have an ICPI of 0.7 or higher (2.0 is maximum). The results listed in Table 1 demonstrated that aHB is an avirulent strain, whereas rHB exhibits a pathogenicity similar to that of the parental virus strain HB. The MDT was 48 h and 56 h for the parental and recombinant rHB viruses, respectively. Moreover, the MDT of the recombinant aHB virus was >120 h, while the ICPI values were 1.92, 1.89, and 0 for the parental virus, recombinant rHB virus, and recombinant aHB virus, respectively.

The newly generated viruses rHB and aHB exhibited titers of 10^8.2^ EID_50_/mL and 10^9.0^ EID_50_/mL, respectively. Thus, the aHB strain exhibited a stronger proliferation capacity in chicken embryos, demonstrating a significant advantage as a candidate vaccine strain.

**Figure 6:**
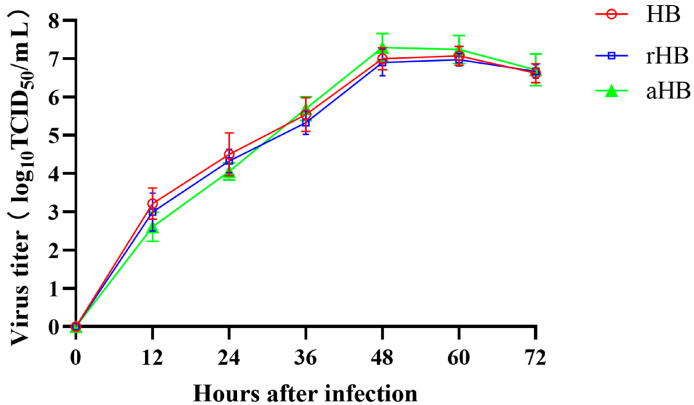
Growth kinetics of the recombinant viruses. Recombinant and parental viruses were used to infect DF-1 cells at an MOI of 0.01. Viral titers were assessed after collecting virus lysates at 12 h intervals for a total of 72 h.

Growth characteristics of the recombinant viruses were examined by inoculating them into DF-1 cells and measuring viral replication at various time points. The replication kinetics of the recombinant viruses rHB and aHB in DF-1 cells were similar to those of the parental strains, reaching peak levels 48–60 h post-infection (Figure 6). The 50% tissue culture infective dose (TCID_50_) of recombinant virus aHB was marginally greater than that of recombinant virus rHB and the parental strain, indicating that the attenuated aHB strain proliferated more effectively in DF-1 cells than it did in the virulent strain, thereby aligning with the proliferation efficiency observed in recombinant viruses within chicken embryos.

The recombinant viruses rHB and aHB were consecutively passaged 10 times in SPF chicken embryos. RNA was extracted from the allantoic fluid, and RT-PCR was used to sequence the genetic markers and cleavage sites of the F protein. Sequencing results demonstrated that the recombinant viruses maintained their genetic stability after 10 passages, as their genetic sequences remained unchanged.

### 3.6. Immunoprotective Effect of the aHB-Inactivated Vaccine

**Figure 7:**
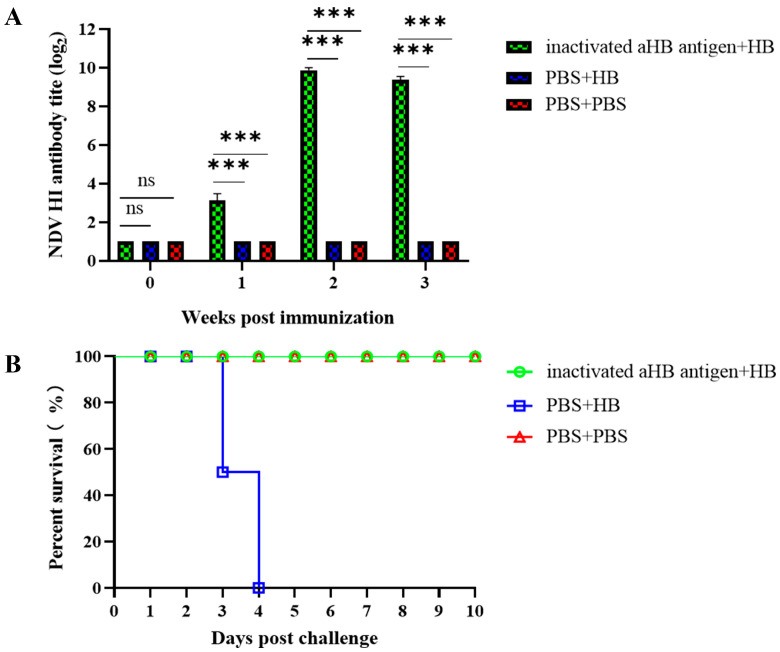
Evaluation of immunoprotective efficacy of the aHB-inactivated vaccine. (**A**) Evaluation of serum hemagglutination inhibition titers following vaccination. Group 1 was immunized with the aHB-inactivated vaccine, while Group 3 received PBS as the negative control group (***, *p* < 0.001; ns, *p* > 0.05). (**B**) Survival status of the chickens after challenge. Groups 1 and 2 were inoculated with the HB strain, while Group 3 received PBS as the negative control.

The inactivated aHB antigen induced a low level of HI titer (3.1 log_2_) 7 days post-immunization, reaching a high level (9.8 log_2_) at 14 days and remaining elevated (9.3 log_2_) at 21 days (Figure 7A). Following the HB strain challenge, group 1 (inactivated aHB antigen + HB) exhibited no clinical symptoms, and all chickens survived until the end of the observation period. Conversely, group 2 (PBS + HB) exhibited reduced feed intake and lethargy on day 3, with partial mortality. All the remaining chickens died by day 4 (Figure 7B). Virus shedding tests revealed no detectable shedding in Group 1 (inactivated aHB antigen + HB) post-challenge (Table 2), indicating that the inactivated aHB antigen effectively prevented viral shedding following challenge with a circulating HB strain.

## 4. Discussions

ND has been present for nearly a century, and at least four global pandemics of the disease have occurred [41]. Since the fourth ND pandemic, the dominant strain in China has been the genotype VII NDV. Most strains are classified under subgenotype VII.1.1, which includes the original VIIb, VIId, VIIe, VIIj, and VIIl [25]. We isolated an NDV strain, identified as genotype VII, and designated it as the HB strain. Comparative genomic analysis of the HB strain with other strains revealed that it belongs to subgenotype VII.1.1 and exhibits high homology with the vaccine strains ZJ1 and SG10 (Figure 2). Research indicates that domestic NDV strains originated in East China and have undergone interspecies transmission and genetic evolution among chickens, geese, pigeons, and ducks [25]. The HB strain demonstrated 82.1–85.1% similarity with the *F* and *HN* genes of the traditional vaccine strains LaSota and B1 and 88.3–99.6% similarity with genotype VII strains. Therefore, there were significant differences in the virulence genes between the epidemic and conventional strains, which may explain why conventional vaccines could not fully prevent the challenge of epidemic strains.

Currently, reverse genetics is among the most widely used techniques for studying NDV pathogenic mechanisms and developing novel vaccines. Here, we developed a reverse genetics platform for the HB strain using the T7 promoter system, incorporating both minigenome and virus rescue systems. To verify the minigenome system, we compared the helper plasmids of the LaSota and HB strains. Interestingly, under the experimental conditions of this study, the functional activity of the helper plasmid from the LaSota strain was superior to that of the helper plasmid from the HB strain. The helper plasmid from the LaSota strain significantly enhanced fluorescent expression of the minigenome system. Therefore, a reverse genetics system incorporating the helper plasmid of the LaSota strain may exhibit increased rescue efficiency, which could greatly facilitate viral rescue efforts.

Helper plasmids can be cross-utilized, and the cross-use of helper plasmids between different strains may enhance the efficiency of virus rescue. Polymerase complex proteins from a single virus can facilitate the replication of closely related viruses within the same genus [42,43,44]. Elbehairy et al. successfully rescued the APMV-3 Wisc using an exogenous *L* gene with the helper plasmid pcDNA3.1-L, employing the *L* gene from APMV-3 Neth. Interestingly, they could not rescue the APMV-3 Wisc virus using their own *L* genes. Nevertheless, replacing the *NP* and *P* genes in the helper plasmid with those from APMV-3 Neth resulted in more effective virus rescue [45]. Notably, APMV-3 Neth replicated more rapidly than did APMV-3 Wisc, potentially explaining why the helper plasmid from APMV-3 Neth more effectively rescued the APMV-3 Wisc strain [46]. Creating a reverse genetics system for paramyxoviruses is difficult, and certain APMVs are not amenable to viral rescue. The unsuccessful rescue of these viruses may be related to the functional activity of the helper plasmids, particularly the L protein. A highly functional L protein may be the key to successful viral rescue. The NDV LaSota strain exhibited good replication capacity, and its L protein may possess better functional activity. Compared with the HB strain, the helper plasmid of the LaSota strain demonstrated better functional activity, rescuing more green fluorescence after transfection. Using this helper plasmid, we successfully obtained recombinant viruses rHB and aHB.

The cleavage site sequence of the NDV F protein is key in determining NDV virulence [47]. Here, aHB, a virulency-reduced strain of HB, was successfully rescued by replacing the cleavage site sequence of the F protein in strain HB (^112^R-R-Q-K-R↓F^117^) with that of the naturally attenuated strain LaSota (^112^G-R-Q-G-R↓L^117^). The ICPI value for aHB was 0, and the MDT value was >120 h. As expected, the aHB strain showed significant attenuation, confirming its safety as a candidate vaccine. The EID_50_/mL of aHB reached 10^9^, which was approximately one order of magnitude higher than that of the HB and rHB strains. This may be because virulent viruses rapidly kill chicken embryos after infection, resulting in a short time for the virus to replicate. Additionally, the attenuated viruses replicate for longer post-embryo infection periods, leading to increased titers. As a vaccine candidate, the high replication level of the recombinant aHB virus in chicken embryos is a significant advantage, as it allows for the easy production of high-titer viruses using chicken embryos. The recombinant aHB virus exhibited a higher titer in DF-1 cells than did virulent viruses. Unlike virulent HB and rHB viruses, the recombinant aHB virus cannot induce syncytial lesions in cells. Similar findings have been previously reported, with the genotype VII-attenuated strains mNA-1 and Ban/AF losing their ability to induce cell fusion [48,49]. The F protein cleavage sites of the aHB and LaSota strains were identical; however, the LaSota strain induced cytopathic effects in cells with the assistance of TPCK-treated trypsin. Therefore, the F protein cleavage site is not the only factor influencing the ability to induce cell fusion.

When the genotype of the vaccine is mismatched with that of the circulating virus, it can only reduce mortality in chickens infected with the circulating virus but cannot completely prevent viral shedding [50]. Genotype-matched vaccines are effective in the prevention of viral shedding and can provide satisfactory immune protection in the context of multivalent vaccines [51]. The generation of vaccine strains with genotypes that align with circulating viruses using reverse genetics systems has become common practice [52]. Notably, the genotype VII NDV strain is virulent, and even when attenuated through reverse genetics, it may revert to virulence due to pathogen gene recombination. Here, we prepared an inactivated vaccine from an aHB-attenuated strain and evaluated its immunogenicity, thereby eliminating the risk of viral reversion to virulence. When challenged with the HB strain of NDV genotype VII, the aHB-inactivated vaccine provided 100% protection to chickens and effectively prevented viral shedding. Thus, recombinant aHB may serve as an effective vaccine candidate.

In summary, we established a reverse genetics operating system targeting a genotype VII virulent HB strain using a helper plasmid from the LaSota strain. Using this system, the genotype VII-attenuated strain, aHB, was produced. aHB has a low virulence, strong proliferation capacity, and excellent immunoprotective efficacy, making it a potential vaccine candidate.

## Figures and Tables

**Table 1:** Biological characteristics of the parental and recovered viruses.

Virus	Pathogenicity		Allantoic Fluids Titer	
	MDT	ICPI	EID_50_	TCID_50_
HB	48 h	1.92	10^−8.25^/mL	10^−7.20^/mL
rHB	56 h	1.89	10^−8.15^/mL	10^−7.20^/mL
aHB	>120 h	0.00	10^−9.0^/mL	10^−7.50^/mL

**Table 2:** Frequency of isolation of challenge virus in chickens.

Groups	Post-Challenge Samples (No. Positive/Total)							
	Day 3		Day 5		Day 7		Day 10	
	O ^a^	C ^b^	O	C	O	C	O	C
aHB + HB ^d^	0/10	0/10	0/10	0/10	0/10	0/10	0/10	0/10
PBS + HB	10/10	10/10	NS ^c^	NS	NS	NS	NS	NS
PBS + PBS	0/10	0/10	0/10	0/10	0/10	0/10	0/10	0/10

After challenge with HB strain, oropharyngeal and cloacal swabs were collected on days 3, 5, 7, and 10. Samples were processed and inoculated into 10-day-old SPF chicken embryos, with allantoic fluid collected after 48 h. Viral replication was determined by the HA assay. Samples were considered positive if HA activity was detected in the allantoic fluid (HA > 2). a: oropharyngeal swabs. b: cloacal swabs. c: no survivors. d: challenged with HB strain.

## Data Availability

The original contributions presented in this study are included in the article; further inquiries may be directed to the corresponding author.

## Supplementary Materials

The following supporting information can be downloaded at: https://www.mdpi.com/article/10.3390/v17121618/s1, Table S1: Primers used for amplification of full-length genomic cDNA of NDV HB strain; Table S2: Primers used for identify the F protein cleavage site sequence; Table S3: Primers for identifying three knocked-out restriction sites within the L gene; Sequence: The full-length genome sequence of the HB strain.
