# Supplementary material for: An Attenuated Recombinant Newcastle Disease Virus of Genotype VII Generated by Reverse Genetics
Source: Viruses. 2025 Dec 15;17(12):1618. doi: 10.3390/v17121618 (PMC12737340; doi:10.3390/v17121618)
Supplement: Supplementary file 1 [file viruses-17-01618-s001.zip › viruses-4013746-supplementary.pdf]

Table S1. Primers used for amplification of full-length genomic cDNA of NDV HB strain.

Primers	sequence (5′-3′)	Size(bp)
FragmentA-F	ACCAAACAGAGAATCTGTGAGGTACGATAAAAGG	2913
FragmentA-R	CAGACTTAATTAATTCAGAGGGGTGCTGCACCGGTTG	
FragmentB-F	CTGAATTAATTAAGTCTGCCACTGCAAGCGGGCC	1681
FragmentB-R	GATACGCGTAGAAGGTTCGGAGCCCATATTG	
FragmentC-F	CTACGCGTATCCCAGCACCTCTGATGCTG	3479
FragmentC-R	GACAATAAGCTTTATTGGTCTTGACAAC	
FragmentD-F	CAATAAAGCTTATTGTCTTAG	3711
FragmentD-R	CTGCTAGCGTCAGCGAGCAC	
FragmentE-F	GACGCTAGCAGATTATGCCC	3456
FragmentE-R	ACCAAACAGAGATTTGGTGAATGACATAAC	

Table S2. Primers used for identify the F protein cleavage site sequence.

Primers	sequence (5′-3′)	Size(bp)
Forward primer	CCCGAATCACCATTACACCAG	1005
Reverse primer	CCCGACTGAGGGCAAATTC	

Table S3. Primers for identifying three knocked-out restriction sites within the L gene.

Primers	sequence (5′-3′)	Size(bp)
MluI-F	TTAAGGATGGTAACCTCAAA	1078
MluI-R	CGAAGCAAACCTTACTCAGTG	
KpnI+HindIII-F	GGTGTGCAAGACATGGGAAC	910
KpnI+HindIII-R	GACCGAATGACTGTGTCAATG	

The full-length genome sequence of the HB strain.

accaaacagagaatctgtgaggtacgataaaaggcgaagaagcaatcgagatcgtacgggtagaaggtgtgaaccccgagcgcgaggccgaag
ctcgaacctgagggaaccttctaccgatatgtcgtctgttttcgacgaatacgagcagctcctcgctgctcagacccgccctaacggaactcatggag
ggggagagaaagggagcactttaaaagttgaggtcccagtatttaccctaaacagtgatgatccagaggatagatggaattttgcggtattctgtcttc
ggattgctgttagcgaggatgccaacaaaccactcaggcaaggtgctcttatatccctcttatgctcccattctcaggtgatgagaaaccatgttgccctt
gcagggaaacagaatgaggccacactggctgttcttgagatcgatggttttgctaacagtgtgccccagttcaacaataggagtggagtgcctgagg
agagagcacagagattcatggtaatcgcaggatctctccctcgggcatgcagcaacggtactccgtttgtcacggctggggttgaagatgatgcacc
agaagatataactgacactctggaaagaatcctatctatccaagttcaggtatgggtcacagtagcaaaggccatgactgcatatgagacagcagatg
agtcagaaacaagaagaataaataagtatatgcagcaaggtcgagttcagaagaagtacatccttcatcctgtatgcaggagtgcaattcaactcaca
atcagacattctctggcagtccgtattttcctagttagtgagctcaagaggggccgcaatacagcaggtgggagctctacatattacaacttggtcggg
gatgtagactcatacatcaggaacaccgggcttactgcatttttcctaacactcaaatatggaatcaataccaagacgtcagccctcgcactcagcagc
ctcacaggtgatatccaaaaaatgaaacagctcatgcgtttatatcggatgaaaggtgaaaatgcaccatacatgacattgttaggtgacagtgaccag
atgagctttgcaccagctgagtatgcacaactttattcttttgccatgggcatggcatcagtcttagataagggaactggcaagtaccaattcgccaggg
actttatgagcacatcattctggagacttggagtagagtatgctcaggcccagggaagtagcattaatgaggacatggctgctgagctaaaactaacc
ccggcagcaaggagaggcctggcagctgctgcccaacgagtatccgaagaaatcggcagcatggacattcccactcaacaggcgggagtcctca

ccgggctcagtgacgaaggcccccgaactccacagggcggatcaaacaagccgcaaaggcaaccagatgccggggatggggagactcaactc
ctggattttatgagagcagtggcgaacagcatgcgggaagcgccaaatcctgcacagagcaccacccatccagagcctcccccaacccctggggc
atcccaagacaacgacactgactgggggtactgatcgacaacacccagcctgccttcacaggatcacaccaaaccctccgcccaaacccctcccac
actccccgacccacaaccccgcacgaccacaccaacaaaagctccccccaccctctcccccacccccagccacacgatcccacccacccggga
caacacaggcacagctcggctcgtcgacaatccgcccagagcccaaggtattagaaaaaaatacgggtagaagagagacatccagagaccagga
cgagtcaccaagttctctgttctcccttctacccagtgaattagggtgaggatggccacttttacagatgcggagatagatgacatatttgagaccagtg
ggactgtcattgacagcataattacggcccagggcaaatcagctgagaccgttggaagaagcgcgatcccgcagggcaagaccaaagctctaagc
acagcatgggagaagcacgggagtgtccagccacacgccagtcaggacgcccctgaccaacaagacagaacagaaaaacagccatccacacct
gagcaggcgactctacacaacaacccgccgatcacatccgcagaaccgcctcccactcaggccgcaagcgagaccagcgacacacagctcaag
actggggcaagcaactcccttctgtccatgctcgacaaactgagcaataaatcgtccaatgctaaaaagggcccatggtcgggttcccaagaagggc
atcaccaacctccggcccaacaacacgggaaccagccgagctatggaagcaaccagggaagaccgcagcatcaggccaaggccgtccctggaa
accggggcacagacgagaacacagcatatcatggacaacggaaggagtcacaaccatcagctggtgcaacccctcatgcgccccagtcagggca
gagccaagacaatactcctgtacctgtggatcgtgtccagctacctgccgactttgcgcaggcgatgatgtctatgatggaggcattatcacagaaggt
aagtaaagttgatcatcagctggacctagtcttgaaacagacatcctccattcctatgatgcgatctgaaatccaacagctcaagacatctgttgcgatca
tggaagctaacttaggcatgatgaaaattctggaccctggttgtgctaacgtttcatccttaagtgatctccgggcagtagcccgatcccacccagtcct
agtttcaggccccggagacccatctccttacgtgacacaagggggtgaaatgacgctcaataaactctcacaaccggtgcagcacccctctgaattg
attaagtctgccactgcaagcgggcctgacatgggagtggagaaggacactgtccgcgcattaatcacctcgcgcccgatgcatccaagctcctcg
gctaggctcctgagcaagctagatgcagccaggtcaattgaagagatcaggaagatcaaacgccttgcgctgaatggttgatggccatcacaactca
taacaggctcccgtcactttagcgtcacacggaatccctcgggggccccccttcgcaaatctatgcttcaacacccaaaacaacagccctctctcacc
ccccccaatcccccgaatgatcgcacaactgcaaccaatccagtggcattagaaattaagaaaaaatacgggtagaatcaaagtgccttgattgcacc
aaaatggactcatccaggacaatcgggctgtactttgattctgccctcccttccagcagcctgttagcatttccgattgtcttacaagacacaggagacg
ggaagaagcatatcaccccacaatacaggatccagcgtcttgattcgtggacagacagtaaggaagactcggtattcatcaccacctacgggttcatc
tttcaaattgggaatgaagaagccaccgtcggtgtgatcaatgacaatcccaggcacgagctactctcttccgcaatgctctgcttagggagtgtcccg
aacgatggagatcttgttgagctggcgagagcctgcctcaccatggtggtaacttgcaagaagagtgcaactaacactgagagaatagtcttctcagt
agtgcaggcacctcgggtgctgcaaagctgtatggttgtggcaaataggtactcatcagtgaatgcagtgaagcatgtgaaggcgccagaaaagatc
cctgggagcggaaccctagagtataaagtgaattttgtctctttgaccgtggtgccaagaagggatgtctacaggatcccaaatgcagtattgaaagtg
tctggctcaagcctgtacaatcttgcgctcaatgtcactattgatgtggacgtggatccgaagagcccgttagtcaaatccctttccaagtccgatagcg
gatactatgcgaatctttttctgcatatcgggcttatgtccactgtagataagaaaggaaagaaagtgacatttgacaagatagaggaaaagataagga
gactcaatctatctgtcgggctcagtgatgtgctcggaccctctgtgcttgtgaaggcgagaggtgcacggactaagctacttgctcctttcttctctagc
agtgggacagcctgctatcctatagcaaatgcctctccccaggttgccaagatactctggagccaaactgcgcacctgcggagtgtgaaagtcatcat
tcaagccggcactcagcgtgctgtcgcagtgaccgctgatcatgaggtaacctccactaagatagagaggaggcatgccattgctaaatacaatcctt
tcaggaaataagttgcatccctaagactgcagttcacctgctttcccgaatcaccattacaccagacaatgatccatctcgactgcttatagttagttcacc
tgcctagcaaattagaaaaaacacgggtagaagagtctggatcccgaccggcacattcaggacgcaatatgggctccgaaccttctaccaggatccc
agcacctctgatgctgatcacccggattatgctgatattgggctgtatccgtccgacaagctctcttgacggcaggcctcttgcagctgcaggaattgta
gtaacaggagataaggcagtcaatgtatacacctcatctcagacagggtcaatcatagtcaagttgctcccgaatatgcccagggataaggaggcgt
gtgcgaaagccccattagaggcatataacagaacactgactactttgctcactcctcttggcgactccatccgcaagatccaagggtctgtgtccacgt
ctggaggaaggagacaaaaacgctttataggtgctgttattggcagtgtagcccttggggttgcaacagcggcacagataacagcagctgcggccct
aatacaagccaaccagaatgctgccaacatcctccggcttaaggagagcattgctgcaaccaatgaagctgtgcatgaagtcaccgacggattatca
caactatcagtggcagttgggaagatgcagcagtttgtcaatgaccagtttaataatacggcgcgagaattggactgtataaaaatcacacaacaggtt
ggtgtagaactcaacctatacctaactgaattgactacagtattcgggccacagatcacctcccctgcattaactcagctgaccatccaggcactttata
atttagctggtggcaatatggattacttattaactaagttaggtatagggaacaatcaactcagctcattaattggtagcggcctgatcactggttaccctat
actgtatgactcacagactcaactcttgggcatacaagtgaatttgccctcagtcgggaacttaaataatatgcgtgccacctatttggagaccttatctgt
aagtacaaccaaaggatatgcctcagcacttgtcccgaaagtagtgacacaagtcggttctgtgatagaagagcttgacacctcatactgtatagagtc
cgatctggatttatattgtactagaatagtgacattccccatgtccccaggtatttattcctgtttgagcggcaacacatcagcttgcatgtattcaaagact
gaaggcgcactcactacgccgtatatggcccttaaaggctcagttattgccaattgtaagataacaacatgtagatgtacagaccctcctggtatcatat
cgcaaaattatggagaagctgtatccctgatagatagacattcgtgcaatgtcttatcattagacgggataactctgaggctcagtggggaatttgatgc
aacttatcaaaagaacatctcaatactagattctcaagtcatcgtgacaggcaatcttgatatatcaactgaacttggaaacgtcaacaattcaatcagca
atgccttggataggttggcagaaagcaacagcaagctagaaaaagtcaatgtcagactaaccagcacatctgctctcattacctatattgttctaactgt
catttctctagttttcggtgcacttagtctgggtttagcgtgttacctgatgtacaaacagaaggcacaacaaaagaccttgctatggcttgggaataatac

cctcgatcagatgagagccactacaagagcatgaatgcagataagaggtggatatatacccaacagcagcctgtgtgtcaattccgataacctgtcaa
gtagaagacttaagaaaaaactactgggaacaagcaaccaaagagcaatacacgggtagaacggtcagaggagccacccttcaatcggaaactag
gcttcacaacatccgttctaccacatcaccaacaacaagagtcaatcatggaccgcgcggtcaacagagtcgtgctggagaatgaggaaagagaag
caaagaacacatggcgcctggttttccggatcgcagtcttacttttaatggtaatgactctagctatctccgcagctgccctggtatacagtacgggggc
cagtacgccgcacgacctcgcaggcatatcgactgtgatctccaagacagaggataaggttacgtctttactcagttcgagtcaagatgtgatagatag
gatatacaagcaggtggctcttgaatccccgctggcgctactaaacactgaatctataattatgaatgcaataacctctctttcttatcaaattaacggggc
tgcgaacaatagcggatgtggggcgcctgttcatgacccagattatatcgaggggataggcaaagaactcatagtggacgacatcagtgatgtcaca
tcattttatccttctgcatatcaagaacacttgaatttcatcccggcgcctactacaggatccggttgcactcggataccctcatttgacatgagcaccacc
cattattgttatactcacaatgtgatactatccggttgcagagatcactcacactcacatcaatacttagcacttggtgtgcttcggacatctgcaacaggg
agggtattcttttctactctgcgctccatcaatttagatgacacccaaaatcggaagtcctgcagtgtgagtgcaacccctttaggttgtgatatgctgtgct
ctaaggtcacagggactgaagaggaggattacaagtcaattgcccccacatcaatggtgcacggaaggctagggtttgacggtcaataccatgaga
aggacttagacaccacggtcttatttaaggattgggtggcaaattacccaggagcgggaggagggtcttttattgacgaccgtgtatggttcccagttta
cggagggctcaaacccaattcacccagtgacactgcacaagaagggaaatatgtaatatacaagcgccataacaacacatgccccgatgaacaaga
ttaccaaattcggatggctaagtcttcatataaacccgggcgatttggtggaaagcgcgtacagcaagccatcttatccatcaaagtgtcaacatccttg
ggtaaggacccggtgctgactattccacctaatacaatcacactcatgggagccgaaggcagaatcctcacagtagggacatctcacttcttgtacca
acgagggtcttcatatttctcccctgccttattatatcccatgacagtaaataacaaaacggctacactccatagtccttatacgtttaatgctttcactcggc
caggtagtgtcccttgccaggcatcagcaagatgccccaactcatgcatcactggggtctatactgatccatatcccttaatcttccataggaatcatact
ctacgaggggtcttcgggacgatgcttgatgatgaacaagcgagacttaaccccgtatctgcagtattcgacaacatatcccgcagtcgtgtcacccg
ggtgagttcaagcagcaccaaggcagcatacacgacatcgacatgttttaaagttgtcaagaccaataaagcttattgtcttagtattgcagaaatatcc
aataccctattcggggaatttaggatcgttcccttattagttgagatcctcaaggatgatagagtttaagaagctagacttggccgattgagccaatcata
ggatggttgggaagacgacaccgcaccaatcatctcccacaatgcttagagtcaagctgaatattaacataagccaggatcccatgttgttgggcagc
cacaatcagacaatgctgacatgattattctgagtcccgcccactatcactttattaagaaaaaatacaaaaagcattgagatataagggaaaacaacca
acaagagggaacacgggtaggacatggcgggctccggtcccgaaagggcagagcaccagatcatcctaccagagtcacatctatcctctccattg
gtcaagcacaaattgctatactactggaaattgactgggctaccgcttcctgatgaatgcgactttgatcatctcattatcagcaggcaatggaagagaa
tactggagtcggccactcctgacacagagagaatgataaaactcgggcgggcagtgcaccagactctcaaccacaattccaagataaccggagtgc
tccatcccaggtgtttagaagaactggctagtattgaggtcccagattcaactaacaaattccggaagattgaaaagaagatccagattcacaacacaa
ggtatggagacctgttcacaaagctgtgcacgcatgttgagaagaaattgctaggatcatcccggtctaataatgtcccacgatcagaggaattcagta
gcatctgtacagatccggcattctggtttcactcaaaatggtccagagccaagttcgcgtggctccatataaaacaagtccaaaggcatctgattgtagc
agcaaggacaaggtctgcagtcaacaagttagtaacattaagtcataagataggccacgtctttgttactcctgagcttgtcattgtgacacatacagat
gagaacaagttcacatgcctcacccaggaactcgtattgatgtatgcggatatgatggaaggcagggacatggtcaatataatatcttctacagcagca
catctcagaaacctatccgagaaaattgatgatattctgcgattagtagatgccctggcaaaggacttaggtaatcaagtctatgacgttgtagcattaat
ggagggattcgcatacggtgctgttcagctgcttgagccatcaggtacatttgcaggagatttctttgcatttaacctacaggagctcaaagacactttaa
tcgaacttctcccaaataatatagcggaatcagtaactcacgctattgccactgtattctccggcttagaacagaatcaagcagctgagatgttgtgcttg
ctacgtttgtggggtcatccattgcttgagtctcgtagtgcagcaagagcagtcaggagccaaatgtgcgcaccaaagatggtagacttcgatatgatc
ctccaggtattatctttctttaaaggaacaatcatcaatggatacagaaagaagaattcaggtgtgtggccgcgtgtcaaagtagatacaatatacggga
atatcattgggcagctgcatgctgattcagcagagatctcacatgatgtcatgttgagggagtacaagagtttatctgctcttgaatttgagccatgtatag
attatgaccctgttaccaatctaagcatgttcctaaaagacaaggcaatcgcacatcctagtgataattggctcgcctcatttaggcggaacctactctct
gaggaccagaagaaacagataaaagaggcaacttcaactaaccgcctcctgatagagttcttagaatcaaatgattttgatccatataaagaaatggaa
tacctgacaaccctcgagtacctaagagatgacagtgtggcagtatcgtactcactcaaagagaaagaggtgaaagtgaatgggcggatttttgctaa
gttaacaaagaaactaaggaactgccaggtaatggcagaaggaattctagctgaccagattgcacctttctttcagggaaatggggtcattcaagatag
catatccttgacaaagagtatgttagcgatgagtcaactgtcctttaacagcaataagaaacgtatcactgactgcaaagagagggtttcctcgaaccg
caatcacgatcagaagagcaagaatcgtagaagagttgccacttttatcacgactgacctacaaaagtattgtcttaactggagatatcagacagtcaa
actattcgcccatgctatcaatcagctgatgggcctacctcatttctttgagtggattcatcttaggctgatggacactacaatgtttgtaggggatcctttc
aatcctccaagtgacccgactgactgtgatctatcaagagtcccaaatgatgacatatatattgtcagtgctagagggggcattgagggactctgccag
aagctatggacgatgatctcaattgctgcaatccaacttgctgcagcaagatctcattgtcgagttgcctgcatggtacaaggtgacaatcaagtaatag
ctgtaacgagagaggtaagatcagatgattccccggatatggtgttgacgcagttgcatcaagctagtgataatttcttcaaggaattgattcatgtcaat
catttgattggccataacctgaaggatcgtgaaaccattagatcagacacattcttcatatacagcaaacgaatattcaaagatggagcaatactcagtc
aggtcctcaaaaattcatctaaattggtgctaatatcaggcgaccttagcgaaaacactgtaatgtcctgtgccaacattgcatctactgtagcacgacta
tgtgagaatgggcttcctaaggatttctgttactatttgaactacctaatgagttgcgtgcagacatactttgattcggagttttctattacccacagctcgca

atcagattccaaccagtcctggatcgaggatatctctttcgtacactcatacgtgttaacccctgctcagctgggggggactgagcaaccttcaatactca
aggctctacacaaggaatattggtgacccagggaccactgctttcgcagaggtcaagcgactagaagcagtggggttgctgagtcccagcatcatga
ctaacatcttaaccaggccacctggcaatggagactgggccagcctatgcaacgacccatactcttttaattttgagactgttgcaagcccaaatattgt
cctcaagaaacatacacagaaagtcctatttgagacatgttcaaaccccttattatccggggtacatacagaggacaatgaggcagaagagaaagcat
tggctgaattcttactcaatcaagaagtgattcacccacgtgtcgcacatgctatcatggaagcaagctctgtgggtaggagaaagcaaattcaagggc
ttgttgacacaacgaacactgtgattaagattgcactgactagggggcccctcggtatcaaaaggctgatgcggataatcagttactcgagcatgcatg
caatgttgttcagagatgatattttcttatccaatagatccaaccacccattagtttcttctaatatgtgctcgctgacgctagcagattatgcccggaacag
aagctggtcacccctgacagggggcaggaaaatactgggtgtatccaaccctgataccatagaacttgtggagggagagattctcagcgtcagtgg
agggtgcacaaaatgtgacagcggagatgagcagtttacttggttccatcttccaagcaatatagagctgactgatgacaccagcaaaaatcccccga
tgagagtgccatatctcgggtcgaagactcaagagaggagagctgcctcgcttgcgaaaatagcccacatgtcaccacatgtgaaagcagcactaa
gggcatcatccgtgttaatctgggcttatggggacaacgaagtgaactggactgctgctcttaatattgcaaggtctcgatgcaacataagctcagagt
atcttcggctattgtcacccctgcccacagctgggaatctccaacatagattggatgatggcataacccagatgacatttacccctgcatctctctacaga
gtgtcgccttacgttcacatatccaatgattctcaaaggctattcaccgaagaaggggtcaaagagggaaacgtggtttaccaacaaattatgctcttgg
gtttatccctaattgaatcactcttcccaatgacagcaaccagaacatatgatgagatcacattacacctccacagtaaatttagctgctgtatccgagaa
gcgcctgttgcggttcccttcgagctcctcgggctggcaccggaattaaggatggtaacctcaaataagttcatgtatgatcctagccctatatcagaga
gagatttcgcgagacttgacttagctatcttcaagagttacgagcttaatttggaatcatattccacgctggagctaatgaacattctttcaatatctagcgg
gaagttgattggccaatccgtggtttcttatgatgaagatacctctataaagaatgatgctataatagtgtatgacaacacacgaaattggattagtgagg
cgcagaactcagatgtggtccgcctgtttgagtatgcagcactcgaagtgctccttgactgtgcttatcaactctactatctgagggtaaggagtctaaa
caacatcgtcctatacatgaatgacttatataagaacatgccagggatcctactctccaatattgcggccacgatatcccaccccatcattcactcaaggt
tgaatgcagtaggcctaattaaccatgacgggtcacaccagcttgcagatatagacttcatcgaggtgtctgcgaaattgttagtctcttgcactcgacg
cgtggtctcaggcttatacgcagggaataagtacgatctgctgtttccatctgtcttagatgataacctgaatgagaagatgcttcaactgatttcccggtt
atgctgtctgtacacagtgctctttgctacaacaagagaaatcccaaaaataaggggcctatcggcagaagagaaatgctcaatactcactgagtatct
actgtcagatgctgtaaaaccattgcttaggcccgaacaagtgagttctatcatttctcccagcataatcacgttcccagccaatctatattacatgtctag
gaagagccttaatttgatcagagaacgagaggacagagatactatcttgtcattgttgttccctcaggaaccactgcttgagcttcgcccagtacgaga
cattggtgctcgagtgaaagacccgtttacccggcaacccgcatcattcatacaaaagctagatctgagtgccccagcaaggtacgacgcatttacac
tgagtaaggtttgcttcgagcatacattaccgaacccaaaggaagattacctagtacggtacttgttcagaggaatagggactgcttcatcttcttggtat
aaggcatctcatcttctatccgtacctgaggtcaggtgtgcaagacatgggaactccttatacttagcggaaggaagcggagccatcatgagtcttcttg
aattgcatataccacatgagactatctattacaatacacttttctcgaatgagatgaaccctccacagcgacatttcggacctacaccaacacagtttctaa
actcggtcgtttataggaatctacaagcggaagtgccatgtaaagatggatatgtccaggagttctgcccattatggagagagaatgcagaagaaagt
gacctgacctcagataaggcagttggatatatcacatctgtggtaccctacaggtctgtatcattactacattgtgacattgagattcctccagggtccaat
caaagcttattagatcaactggctactaatttatccctgattgccatgcattctgtgagggagggcggggtagtgatcatcaaagtactgtatgcaatgg
ggtactacttccatttactcatgaatttattcactccatgttccacgaaaggatatatactctccaatggctatgcctgtagaggggatatggagtgttacct
gatattcgttatgggctacttaggcgggcccaccttcgtgcacgaagtggtaaggatggcaaaaactctaatacaacgacacggtacacttctatctaa
atcagatgaaattacattgactaagctatttacctcacagcagcgtcgtgtaacagatatcctatccagccctttaccgaagctaatgaagctcttgagag
aaaatattgatgctgcactaattgaagccgggggacagcccgtccgtccattctgtgcggaaagtttggtgagcacactaacagatatgacccagaca
acccagatcattgccagccacattgacacagtcattcggtctgtaatttacatggaggctgagggtgacctcgccgacacagtgttcttatttactcctta
caatctatccacagacggtaaaaagagaacatcacttaagcagtgcacaaaacagatcttggaagtcacaatactgggtctcagagccaaagatatca
ataaagtaggtgatgtaatcagtttagtactcagaggtgcggtttccctagaggacctcatcccattaaggacatacctgaagcgcagtacctgccctaa
atacttgaaagcggtcctaggtattactaaactcaaagaaatgttcacagatacctcgttactgtacttgactcgtgctcaacaaaaattctacatgaaaac
cataggtaatgctgccaagggatattacagtaataatgactcttaaaggcaatcgtacgctaatcagttaccttcctaactgatgactccctcactgactta
attataccagattagaaaaaagttaaattccgactctttggaactcgtattcggattcagccagtcaacttcaagcaagagtgcgcaaagtcgcccctaat
catagttatgtcattcaccaaatctctgtttggt
